# Feasibility and Acceptability of a Mobile Technology Intervention to Support Postabortion Care (The FACTS Study Phase II) After Surgical Abortion: User-Centered Design

**DOI:** 10.2196/14558

**Published:** 2019-10-10

**Authors:** Roopan Kaur Gill, Gina Ogilvie, Wendy V Norman, Brian Fitzsimmons, Ciana Maher, Regina Renner

**Affiliations:** 1 Women's Health Research Institute Department of Obstetrics and Gynaecology University of British Columbia Vancouver, BC Canada

**Keywords:** mHealth, abortion, digital health, human centered design, knowledge translation, women's health, sexual health

## Abstract

**Background:**

Human-centered design is a methodology that applies an iterative participatory process that engages the end-user for whom an innovation or intervention is designed for from start to end. There is general evidence to support the use of human-centered design for development of tools to affect health behavior, but specifically for family planning provision. This study is part two of a three-phase study that uses a user-centered design methodology which uses the findings from Phase I to design, develop, and test a digital health solution to support follow-up after an induced surgical abortion.

**Objective:**

The objectives for this study were to: (1) develop a Web-based intervention based on preferences and experiences of women who underwent an abortion as measured in the formative phase of the Feasibility and Acceptability of a Mobile Technology Intervention to Support Postabortion Care Study; (2) conduct usability testing of the intervention to determine user-friendliness and appropriateness of the intervention; and (3) finalize a beta version of the Web-based intervention for pilot testing.

**Methods:**

The study design was based on the “development-evaluation-implementation” process from the Medical Research Council Framework for Complex Medical Interventions. This study is in Phase II of III and is based on user-centered design methodology. Phase I findings demonstrated that women engage with technology to assist in clinical care and they preferred a comprehensive website with email or text notifications to support follow-up care. In Phase II we collaborated with family planning experts and key stakeholders to synthesize evidence from Phase I. With them and a development partner we built a prototype. Usability testing was completed with 9 participants using a validated System Usability Scale. This was then used to refine the intervention for Phase III pilot study. This study was approved by the local Ethics board.

**Results:**

We developed a comprehensive Web-based tool called myPostCare.ca, which includes: Post-Procedure Care, Emotional Well-Being Tool, Contraception Explorer, Sexual Health, Book an Appointment, and Other Resources. Additionally, over the course of a month after the procedure, automatic email notifications were sent to women as a form of virtual follow-up support, directing them to myPostCare.ca resources. The Web-based tool was refined based on usability testing results.

**Conclusions:**

This study demonstrated that user-centered design is a useful methodology to build programs and interventions that are women-centered, specifically for abortion care.

## Introduction

Despite there being no legal restriction to abortion care in Canada, women who seek or have an abortion continue to experience stigma across the country. This has the potential to leave them feeling isolated and unsupported, and potentially prevents them from seeking follow-up care if needed. Therefore, innovative approaches for using information and communication technologies to achieve enhanced health service delivery, broadly known as digital health [1], is a way to address these issues. Digital health interventions in the form of hotlines, text messaging, and mobile applications have been shown to be safe, effective, and acceptable to women and providers for delivery of various aspects of abortion care [2-7]. Ensuring that an innovation is acceptable to the end-user and incorporating their voice throughout the research process is essential. Human-centered design is a methodology that implements an iterative participatory process by applying the needs of the end-users to the development of a given technology solution [8,9]. This methodology has been widely used for the design of innovations that generally affect behavior change.

Digital technology is changing the way we collect information and share and consume data. There is a growing momentum in the provision of resources for family planning, but specifically towards safe abortion care, in terms of the use of digital health interventions to address service delivery and legal barriers in various contexts. The importance of incorporating the end-user perspective’s voice into the design and development of these interventions is crucial, as it has been noted that there are few mobile interventions that are truly effective and scalable [4,9]. Utilizing user-centered design for the development of a mobile tool that women can use to self-manage their care after a surgical abortion will lead to a higher likelihood that it will be acceptable and feasible to use and implemented to scale.

This study is Phase II of III. The findings from Phase I, which are published separately, were essential to Phase II [10]. The main objectives for this study included: (1) the development of a Web-based intervention based on the preferences and experiences of women who underwent an abortion as measured in the formative phase of the Feasibility and Acceptability of a Mobile Technology Intervention to Support Postabortion Care (FACTS) Study; (2) usability testing of the intervention to determine user-friendliness and appropriateness of the intervention; and (3) finalizing a beta-version of the Web-based intervention for pilot testing. Phase III of this three-phase study will determine acceptability and feasibility of the tool in a pilot prospective mixed-methods study. This study is the first in Canada to utilize user-centered design to develop a mobile intervention to support follow-up care after a surgical abortion.

## Methods

### Overview

**Figure 1:**
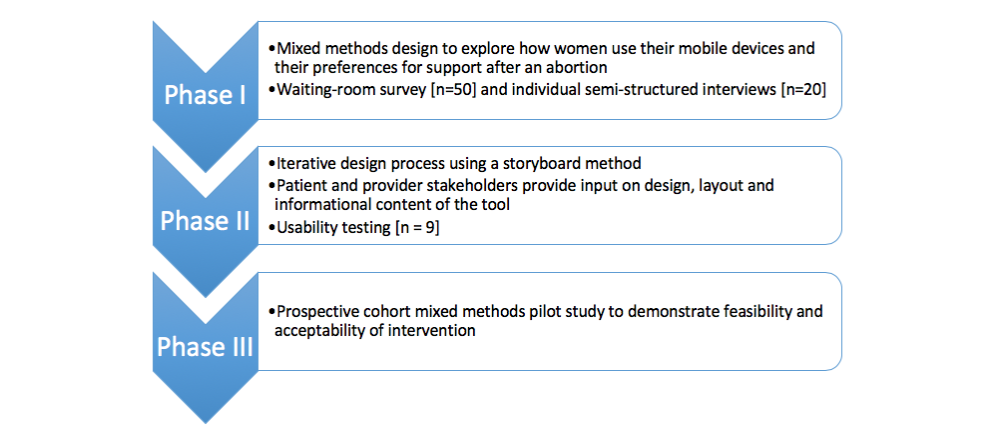
Flow chart depiction of the three-phase study design.

The methods presented below are specific to the design, development, and usability testing of the intervention. A systematic visual depiction of each phase is provided in Figure 1.

### Development and Design

Employing user-centered design, a systematic process was used to develop a mobile intervention based on the results from Phase I. This was conducted from September 2017 to January 2018. User-centered design is a methodology with “roots in a participatory process” and:

provides a framework to understand and apply the needs of end-users to mHealth project development through a highly iterative process [9]

We collaborated with University of British Columbia family planning experts to synthesize evidence and create a storyboard. A storyboard process is used to build a short narrative to visually plot elements of a prototype [8]. This process is allocated 60 minutes per session and uses a series of comic book style frames for drawing and highlighting the narrative of the mobile intervention [8]. The number of sessions is dependent on the complexity of the intervention. This is a useful step in the ideation process of design and development, and design companies use this process as an important early stage of user-centered design for health innovations.

We engaged various stakeholders, using implementation science principles, with the intent to assess the context that the intervention would be potentially applied to. This included stakeholders such as end-users, the hospital administration, funders, health care providers, family planning experts, and donors. In addition, we further engaged key rural stakeholders from Northern Health Prince George Hospital. Specifically, a focus group session was held with four providers from Prince George Hospital. The presentation of results from Phase I and a storyboarding session with the providers highlighted the facilitators and barriers to the provision of safe abortion care in rural British Columbia, particularly regarding follow-up support. These results were used to further refine content and design of the intervention.

By October 2017, an initial prototype for a comprehensive Web-based solution was developed with the support of a development partner and the findings from Phase I [10]. A scope document included the required key features for design and content.

### Theoretical Framework

Like Phase I, the study design for Phase II was informed by the Technology Acceptance Model and the Theory of Reasoned Action [10-12]. Both these theories assess the perceived ease of use and usefulness of a system and individual’s conduct based on their lived experiences, attitudes, and intention to engage in a behavior. As highlighted in Phase I, the study instruments for all three phases were developed using these theories based on validated survey tools [10].

### Usability Testing

Recruitment of usability testing participants initially included contacting women from a database created at one of the abortion clinics in Vancouver that had a list of those who had consented to participating in future research. Due to limited response rates, we proceeded to utilize a social media recruitment strategy through two provincial and national reproductive and sexual health advocacy organizations, Action for Sexual Health Canada and Options for Sexual Health. This included Twitter and Facebook notifications. Eligible participants contacted the research coordinator and received a link to the website, a password and username, and a link to a survey. We did not collect demographic data.

A validated questionnaire adapted from the 2010 Post Study System Usability Questionnaire (PSSUQ) was used to assess participants’ qualitative and quantitative feedback on usefulness, ease of use, privacy and security, content, visual layout, and general concerns [13]. Participants were recruited from a database of women who had consented to be contacted for future research at the CARE Clinic at British Columbia (BC) Women’s Hospital. Participants were also recruited through social media advertising by national reproductive and sexual health organizations that used their respective Twitter accounts to share the link to the study website. Participants who were locally recruited conducted usability testing at BC Women’s Hospital with researchers present. For those recruited through social media, participants received a link to the survey by email and details about how to access the website. A team of key stakeholders made up of obstetrics and gynecology specialists, family doctors, counsellors, nurses, and administrators provided feedback about the initial prototype of the intervention. Participants provided feedback on usefulness, ease of use, privacy and security, content, visual layout, and general concerns.

### Data Analysis

During Phase 2, we performed descriptive data analysis. Results of the PSSUQ survey were reported in percentage (%). An official score was not calculated as the survey was adapted from the PSSUQ but was not used in its entirety. The adapted survey can be found in Multimedia Appendix 1.

This study was approved by the Children’s and Women’s Research Ethics Board (H16-02823).

## Results

### Summary

Phase II participants for the storyboarding process included key stakeholders from Vancouver and Prince George Hospital. The health care providers (HCPs) had a median of 12 years (range: 1-20 years) of experience in family planning. These HCPs included: physicians, counsellors, nurses, and administrators. The development company selected for the study was a local software development group. We conducted 5 storyboarding sessions, which included the following: (1) family planning specialists in Vancouver; (2) rural providers in Prince George Hospital that included one family doctor and three specialist obstetrician/gynecologists; (3) five counsellors from an urban clinic in Vancouver; (4) a participant who had previously had an abortion and volunteered to participate; (5) a session with the investigators of this study; and (6) three senior administrative staff involved with one urban abortion clinic. Each session lasted between 60 to 90 minutes.

### Key Stakeholders Engagement

Based on our stakeholder analysis we developed a communication strategy for engaging them, including the development of a facts sheet about the study, a website explaining the study, and standard presentations. The first step was to meet with each stakeholder and provide an orientation to the concept of a postabortion support tool using mobile technology. This was also an opportunity to further discuss their level of involvement for development and implementation of the intervention. Ongoing updates were provided with in-person meetings, telephone calls, and email bulletins. Table 1 highlights the key stakeholder groups, their respective area of influence or interest, the project phase, the engagement method, and the frequency with which they engaged with the development of myPostCare.ca.

**Table 1:** Key Stakeholder Engagement Matrix.

Stakeholders		Area of influence or interest	Project Phase	Engagement Method	Frequency
**Health Care Practitioner**					
	BC^a^ Abortion Providers (Vancouver and Prince George)	Content advisor Adopter of intervention	All	Presentations Monthly Meetings Story board participants	Monthly
	Family Planning Experts (UBC^b^, UCSF^c^, UCLA^d^)	Content advisor	All	Meetings	Bimonthly or as needed
	Counsellors	Content advisor Patient behavior expert Recruitment Adopter of intervention	Phase I Phase III	Luncheon presentations Recruitment updates Training sessions Feedback opportunities	Monthly
**Researchers**					
	WHRI^e^ Children’s and Women’s Research Ethics Board Family Planning Research Committee	Research administration Project management Provision of ethical standards Provide research support	All	Meetings and Check-ins	Weekly As needed Monthly
**Consumer/End User**					
	Individuals receiving care at 3 urban abortion clinics in Vancouver	Guide content for intervention and user design preferences	Phase I Phase III	Provided honorariums Surveys Over the phone interviews	Weekly during recruitment periods
	Remote participants (Individuals who previously had an abortion procedure)	Guide content for intervention and user design preferences	Phase II	Provided honorariums Online and Face to Face engagement	Weekly during recruitment periods
**Industry**					
	Website/App Developers	Develop Resource Creative Expertise	Phase II Phase III	Face to Face & Online meetings Payment	Weekly
**Technical Experts**					
	PHSA^f^ privacy and security BCCHRI^g^ web services	Ensured website security, safety of participants, and best practice at pilot site	Phase II Phase III	Consulting	Weekly As needed
**Advocacy Groups**					
	Options for Sexual Health Action Canada for Sexual and Reproductive Rights	Assistance with Recruitment Advocates	Phase II	Presentations Grand rounds	Biannual
**Decision Makers**					
	Program Directors Hospital CEO^h^ and COO^i^	Facilitation of research Sustainability	All	Written communication Meetings/ Presentations	Monthly
**Funders**					
	Family Planning Fellowship BC Women’s Hospital Foundation	Finances Sustainability	All	Written communication Meetings/ Presentations	Quarterly As needed

^a^BC: British Columbia.

^b^UBC: University of British Columbia.

^c^UCSF: University of California San Francisco.

^d^UCLA: University of California Los Angeles.

^e^WHRI: Women’s Health Research Institute.

^f^PHSA: Provincial Health Services Authority.

^g^BCCHRI: British Columbia Children’s Hospital Research Institute.

^h^CEO: chief executive officer.

^i^COO: chief operating officer.

### Storyboarding

The formative research findings from Phase I were used to inform the creation of storyboards in collaboration with the research team and family planning experts based at the University of British Columbia. Two storyboards were created: (1) design; and (2) content for the mobile intervention. These storyboards took into consideration information based on the preferences that were elicited from the findings in Phase I [10]. The storyboard was reviewed in an iterative manner by the family planning experts and research team. It was also shared with members of the administration and allied health care providers at the three abortion clinics where recruitment for Phase I was conducted.

### Development

Once the storyboard was completed, this was shared with a design and development company in Vancouver, British Columbia that is an expert in Web-based technologies for social marketing and behavior change and has experience working with the Ministry of Health in British Columbia. A step-by-step process was executed between the developer and the research team to build the prototype for the Web-based tool, which was a website that was accompanied by an email system. This came to be called myPostCare.ca. The steps of the process are highlighted in Table 2. The components of myPostCare.ca are highlighted in Table 3.

**Table 2:** Scope tasks for development of mobile intervention.

Scope Tasks	Description
Discovery Sessions	To discuss and uncover key aspects of website and email notifications to create comprehensive scope of work
Information Architecture	Development of wireframes Create workflow document of user and administrative experiences
Content Review and Copyediting	Development of content by client for all pages Developer to provide recommendations and feedback based on creating cohesive user experience
Design Development	Presentation of design proofs with 2 rounds of revisions
Technical Development	Use of PHP^a^-based content management system (ie, Wordpress) deployed to meet functional requirements Content population with interactive elements Review and revision
Web Analytics	Incorporate secure web analytics into website
Quality Assurance and User Acceptability Testing	Quality assurance and optimization for current versions of industry standard browsers Ensure compatible on various mobile devices (ie, response website) Address system errors and bugs
Deployment and Training	Deployment and hosting to third party company Training of client to provide understanding of editing functionality provided in system

^a^PHP: hypertext preprocessor.

**Table 3:** Structure of myPostCare.ca

Sections		Content References
1. Postprocedure Care		Woman-centered postabortion care: Reference manual. [14] British Columbia Women’s Hospital CARE^a^ Clinic Postprocedure care resources Everywoman’s Health Centre [15]
2. Contraceptive Explorer		
	Interactive patient-centered screen Detailed information about each contraceptive method: effectiveness, cost, hormone free, prevent against STI^b^, access, side effects	CDC^c^/WHO^d^ Medical Eligibility Criteria [16,17] Bedsider [18] Sex & U [19]
3. Emotional Well-Being Tool: How are you feeling today? Responses: Good, Ok, not so Good (Sources provided for specific emotions with definitions, strategies and resources)		Exhale Website [20] Pregnancy Options [21] Peace After Abortion [22] All-Options [23] Decision Assessment and Counseling in Abortion Care: Philosophy & Practice [24] Everywoman’s Health Centre [15] Expert consultation with counsellors from Everywoman’s Health Clinic, British Columbia Women’s CARE Clinic, Elizabeth Bagshaw Clinic, and University of San Francisco
4. Sexual Health		
	Menstrual Cycle Interactive Tool Menstrual Cycle Trackers Sexual Health Resources	Interactive tool content adapted from Williams Gynecology 2^nd^ Edition [25]
5. Book a Counsellor		Not applicable
6. Myths and Facts Interactive Quiz		Willow Clinic [26]
7. Five circulating Articles		
	Meditation 101 Advice for Partners How to talk to Family and Friends FAQ^e^	Meditation developed in partnership with Moment Meditation, local Vancouver meditation centre Everywoman’s Health Centre British Columbia Women’s Hospital CARE Clinic Resource Sheet
Resources		Content developed in collaboration with counsellors at British Columbia Women’s Hospital CARE clinic and Everywoman’s Health Centre
About Us		Not applicable

^a^CARE: Abortion Clinic (CARE program).

^b^STI: sexually transmitted infection.

^c^CDC: Centers for Disease Control and Prevention.

^d^WHO: World Health Organization.

^e^FAQ: frequently asked questions.

### Usability Testing

As stated in the methods, user testing occurred both in person and remotely. Participants were given access to the website and after reviewing it completed an adapted version of the PSSUQ 2010 and provided qualitative feedback. There were 7 remote participants and 2 in-person participants.

The survey results adapted from the PSSUQ 2010 are available in Multimedia Appendix 2. Participants were satisfied with the usability of myPostCare.ca. Specifically, 62.36% “Strongly Agreed” and 28.69% “Somewhat Agreed” with the overall usability of the website. The PSSUQ reflects the overall usability of a website or app based on the respondent’s experience. It has 3 subscores derived from subsets of 16 questions. Overall usability defined by the PSSUQ reflects system usefulness, information quality, and interface quality. Table 4 highlights the comments that participants shared and that were noted in the revisions of the prototype to prepare myPostCare.ca for the Phase III pilot study. Like Phase I, participants were accustomed to using some form of technology and were supportive of a Web-based tool to support follow-up care after an abortion. This was elicited from the key findings from potential users who completed the usability testing.

**Table 4:** Key findings from potential users on the acceptability and perceptions of Web-based sexual health services/testing and how these influenced the design of myPostCare.ca

Subject	Quotes
Emotional Wellbeing Tool	Navigation through the emotional well-being tool was found to be cumbersome, with too many clicks, and difficult navigation back to the most recently page viewed Having accessible drop boxes rather than having to scroll up and down would be helpful Change wording of the term emotional tool–is the tool emotional? Have new articles that would be posted semiregularly, consider having guest articles Consider reordering the recommendations for each emotion as the logic would be different based on whether one is feeling isolated versus relieved Basic and repetitive suggestions on emotional tool
Website Branding or Contact Us section	More clarity on what myPostCare.ca is and what it does, provide more information about the FACTS^a^ team, missions and values, acknowledgements and the funders Reorganize the other resources page and consider organizing according to issue, community, etc Contact us was buried in the about us section; would be helpful to separate this
Postprocedure Care	Reorganize content so “what to expect” and emergency information comes later, or have it as another column next to “precautions”, “pregnancy and periods” to compare/contrast what is to be expected versus what is ER^b^ worthy Consider adding the emotion “Fear” in the emotional well-being tool as this was the feeling I experienced after having my abortion. For instance, feelings that I may bleed out or get an infection. Add more supportive and reassuring content on the landing page, for example comment that abortion is safe, there are supports available to you and you are not alone
Sexual Health Section	Ensure that the links are all working Formatting of the hyperlinks for the menstrual cycle tools and other resources
Contact a Counsellor	Add “Book a Counsellor” to the side bar so that it is more prominent and easier to find Add the helpline information at the bottom of each page so that it is accessible to the user
Contraception	On the sexual health cost page, the lowest cost options are also the least effective, but on the page, they’re presented the same. It reads like a low-cost recommendation for contraception. I wonder if there's some way to display that it's low cost but not effective. For lower income women, seeing this might reinforce cheaper methods and discourage more effective methods. For more expensive methods of contraception, information on any available supports would be helpful. Some health plans cover IUDs^c^ and birth control, for example. Do any clinics or orgs help cover the cost of the pill or IUDs? Overall comparison page would have been useful
Privacy	Need a more clearly stated privacy statement Not sure if a sign in and registration is required of the website; might be useful to have this accessible to all comers
Inclusivity	This site is for women who have had surgical abortions, but it would be useful to include medical abortion to this as well Something that really dictates whether I am a fan of a resource or not is inclusivity. At this point, FACTS seems very heteronormative and cis-centric. It would be nice to see a section dedicated to resources for loved ones, parallel set of “post procedure care” and “emotional well-being sections” for people’s support systems so that they can be informed and feel competent in supporting their loved ones who have undergone an abortion
General Design	Simple layout was easy on the eyes, simple language and openness of tone User friendly, and it covered things I wish I had known after my procedure Made me feel like I am part of a community of people Not overwhelming to use Have featured content visible on other pages aside from home page Easy but there were too many clicks needed to navigate through, making it difficult to navigate More explanation about who has an abortion, what is normal Emotional tool was great, easy to navigate and tips were targeted and useful

^a^FACTS: Feasibility and Acceptability of a Mobile Technology Intervention to Support Postabortion Care.

^b^ER: emergency room.

^c^IUD: intrauterine device.

The email notification system was developed in collaboration with family planning experts, physicians, and counsellors. Their expertise was used to specify what type of messaging would be appropriate at which time interval. This was complimented with results from Phase I of timing and content of email or text messaging. Two email streams were developed: one for participants who had an intrauterine device (IUD) inserted and another for those who did not have an IUD inserted. The emails were sent starting on the day of procedure (day 0) followed by every other day for one week and then weekly until day 28. The first week was focused on post-procedure signs and symptoms, and the next three weeks alternated between contraception counselling, emotional support, and overall sexual health information. The design of the email notifications was aligned with the design of myPostCare.ca. The content was developed by the primary investigator and reviewed by counsellors at the abortion clinics. The messaging was repurposed based on the social marketing expertise of our developer.

## Discussion

### Primary Findings

myPostCare.ca is the first comprehensive Web-based postabortion tool in Canada and has the potential to be integrated as part of family planning services. It includes four interactive tools (Emotional Support Tool, Contraceptive Explorer, Postprocedure Care, and Sexual Health) that integrate automatic email notifications to provide support over the course of one month after the procedure. Integration of myPostCare.ca into clinical practice provides an opportunity to consider a new approach to supplement follow-up care specifically for abortions, but also women’s health in general. We utilized user-centered design methodology, an iterative development process that was informed by input from key stakeholders such as patients, family planning experts, and administrators who are involved with abortion care [27-29]. This was crucial in developing a tool that responded to findings from Phase I [10].

Specifically, this phase demonstrated the importance of including the end users and key stakeholders in the design, development, and testing of a mobile intervention that services a population and deals with a health care issue that continues to be stigmatized. The formative research indicated essential information regarding women’s interactions with technology, their needs and desires around follow-up and access to information, and their feedback on design, which was essential in the success of myPostCare.ca. An iterative design process was important to ensure that the research team was continually evaluating that myPostCare.ca realized the needs of the target users. Similar studies have successfully demonstrated that using this approach leads to a higher likelihood of implementation and scalability [3,27-29].

We adopted a few theoretical frameworks, all of which use a comprehensive participatory approach to developing eHealth technologies. This was similarly done by Gilbert et al in the development of Get Checked Online, which is a Web-based sexually transmitted infection testing resource [29]. More specifically, integrating the Technology Acceptance Model and Theory of Reasoned action with the user-centered design methodology let us use a holistic approach to develop myPostCare.ca. According to the Technology Acceptance Model, perceived ease of use and perceived usefulness of a system are the two predominant indicators of system adoption [11,30]. Participants in our study were accustomed to using some form of technology, either mobile phones or computers, did not require acquisition of new skills, and were keen on the development of a technology-based tool to support follow-up care after an abortion. Importantly, myPostCare.ca will not eliminate structural barriers to comprehensive abortion care, and though it may not directly affect health behavior and decision-making, it may assist with making the delivery of abortion care more efficient, convenient, patient-centered, and accessible.

The limitations for this study include overall generalizability to other populations, small sample size for usability testing, loss to follow up, and recruitment bias. As it pertains to recruitment bias, those who consented to participate were likely individuals who were more engaged with technology, of a higher socioeconomic demographic, and were more likely to be early adopters of a digital health intervention to support abortion care. Though demographic data was not specifically collected for Phase II, this is based on the demographic data collected in Phase I [10]. In previous studies this has been noted as a digital divide, which suggests that though many developers of technology-based health interventions are optimistic about their impact, this needs to be balanced by the fact that the pattern of adoption is along social gradients [29]. New technologies like myPostCare.ca may further reinforce these social divides. Furthermore, abortion continues to be a stigmatized issue, which can be limiting for research since it can be a sensitive topic for most. In our study, it posed difficulties with recruitment and loss to follow-up. We assumed that lack of participant engagement may be associated with stigma about abortion, so we had to reevaluate our usability testing strategy regarding using social media platforms, which proved to be more successful as more participants were willing to engage anonymously at a distance. This recruitment strategy for abortion-specific studies is promising, particularly when thinking about diversifying the participants recruited and obtaining robust response rates for analysis.

Balancing these limitations are the strengths of this study, including: successful development of user-centered design elements, wide stakeholder engagement, diverse expertise on the research team, rigorous research methodologies, iterative design process, and development of the first Web-based postabortion tool in Canada, with the potential to expand it to other aspects of women’s health (eg, miscarriage, gynecologic cancer care, sexual pleasure, and well-being).

Further research to evaluate acceptability and feasibility of myPostCare.ca and overall patient experience will be assessed in a prospective pilot mixed-methods study, which is Phase III of this three-phase study. In addition, as suggested in other Web-based literature [29], a health equity impact assessment with expert consultation and literature review may also help identify ways in which myPostCare.ca reinforces or alleviates health inequities in sexual health services.

### Implications

By using user-centered design and rigorous key stakeholder engagement, there is potential for digital solutions for women’s health to be implemented at scale. This study demonstrated that, by engaging end-users throughout the design of an intervention targeted to them, this provides insights and nuances that have implications for usability, acceptability, and feasibility to integration as a part of clinical care.

## Appendix

Multimedia Appendix 1 Pictorial representation of FACTS (Feasibility and Acceptability of a Mobile Technology Intervention to Support Postabortion Care) three phase study design.

Multimedia Appendix 2 Adapted survey results for usability testing using PSSUQ 2010 overall satisfaction scores reflecting system usefulness, information quality and interface quality. PSSUQ: Post Study System Usability Questionnaire.

## Abbreviations

BC: British Columbia
FACTS: Feasibility and Acceptability of a Mobile Technology Intervention to Support Postabortion Care
HCP: health care provider
IUD: intrauterine device
PSSUQ: Post Study System Usability Questionnaire
