# Supplementary figures and images for: Feasibility and Acceptability of a Mobile Technology Intervention to Support Postabortion Care (The FACTS Study Phase II) After Surgical Abortion: User-Centered Design
Source: JMIR Hum Factors. 2019 Oct 10;6(4):e14558. doi: 10.2196/14558 (PMC6819013; doi:10.2196/14558)

## Multimedia Appendix 1

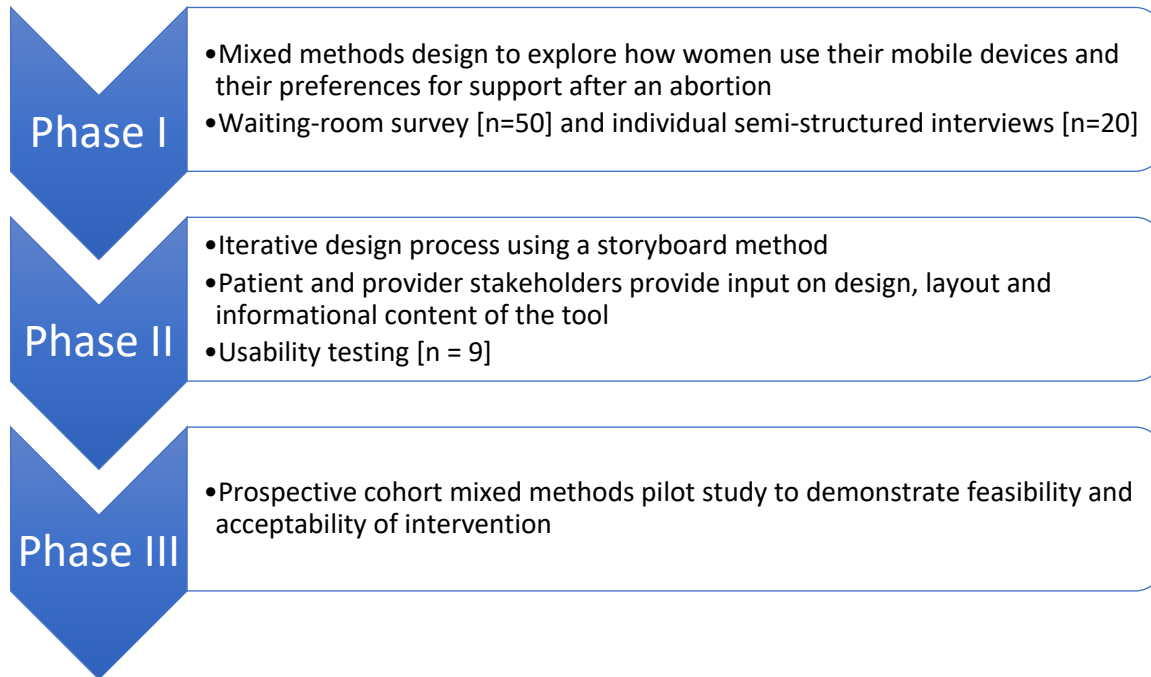

**Figure 1.** Pictorial representation of FACTS three phase study design.

Supplement: Multimedia Appendix 1 [file humanfactors_v6i4e14558_app1.pdf]
