# Supplementary material for: Feasibility and Acceptability of a Mobile Technology Intervention to Support Postabortion Care (The FACTS Study Phase II) After Surgical Abortion: User-Centered Design
Source: JMIR Hum Factors. 2019 Oct 10;6(4):e14558. doi: 10.2196/14558 (PMC6819013; doi:10.2196/14558)
Supplement: Multimedia Appendix 2 [file humanfactors_v6i4e14558_app2.pdf]

# Multimedia Appendix 2

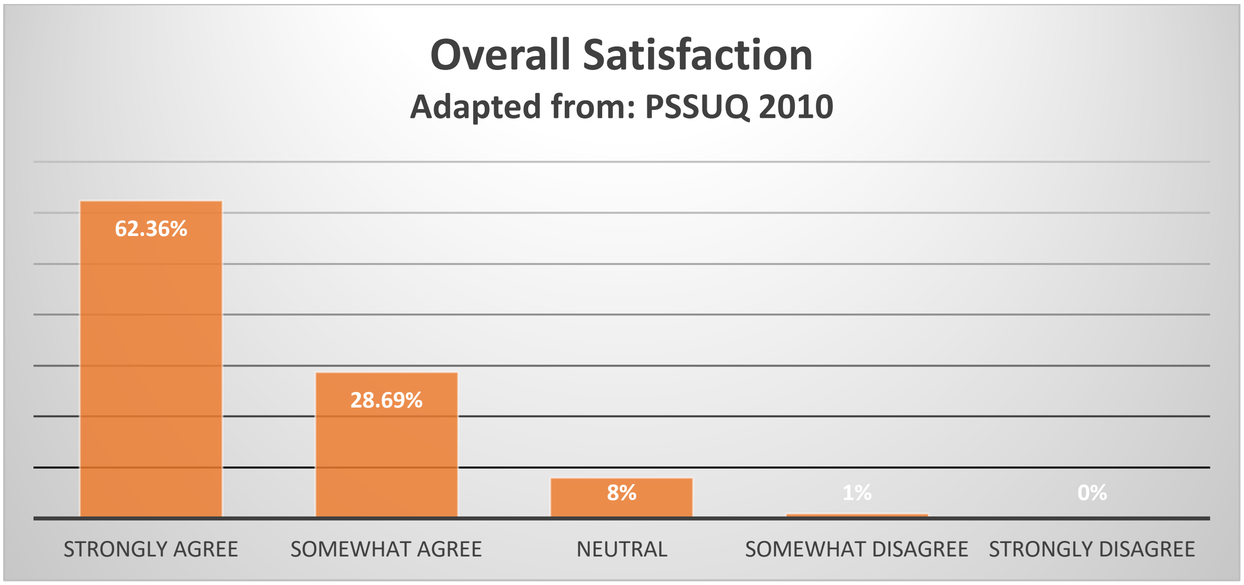


**Figure 2.** Adapted survey results for usability testing using PSSUQ 2010 overall satisfaction scores reflecting system usefulness, information quality and interface quality.
